# Sustained clinical response after single course of rituximab as first-line monotherapy in adult-onset asthma and periocular xanthogranulomas syndrome associated with IgG4-related disease: A case report

**DOI:** 10.1097/MD.0000000000011143

**Published:** 2018-06-29

**Authors:** Giovanni Pomponio, Diletta Olivari, Massimo Mattioli, Alessia Angeletti, Giulia Rossetti, Gaia Goteri, Armando Gabrielli

**Affiliations:** a Clinica Medica, Ospedali Riuniti; b Clinica Medica; c Section of Pathological Anatomy, Università Politecnica delle Marche, Ospedali Riuniti, Ancona, Italy.

**Keywords:** adult-onset asthma and periocular xanthogranulomas syndrome, IgG4-related disease, lacrimal gland, lung mass, parotid, rituximab

## Abstract

**Rationale:**

IgG4-related disease (IgG4-RD) is an emerging immune-mediated disease characterized by multi-organ involvement and variable clinical behavior.

**Patient concerns:**

We describe the case of a 50-year-old woman affected by a rare variant of IgG4-RD, characterized by eyelid xanthelasmas, adult-onset asthma and salivary and lacrimal glands enlargement. Multiple lymphadenopathies and a pulmonary mass were present at initial evaluation.

**Inteventions:**

After a single course of rituximab (2g in 2 refracted doses), an almost complete clinical remission was achieved without chronic steroid administration.

**Outcomes:**

Magnetic resonance imaging (MRI), high-resolution computed tomography (HRCT) of the thorax, and positron emission tomography (18FDG-PET-CT) confirmed good response to treatment. Circulating plasmablasts dropped to undetectable levels as well. Xanthelasmas only remained unchanged. Remission persisted at 1-year follow-up.

**Lessons:**

Steroid therapy is still considered standard first-line therapy in IgG4-RD. However, high doses are generally required and relapses are common during the tapering phase. Rituximab is a well described steroid-sparing strategy, so far reserved to refractory cases only. In our experience, rituximab has been used as first-line monotherapy, showing great and sustained efficacy and optimal tolerability. The peculiar variant of IgG4-RD affecting our patient, the relatively low baseline plasmablast concentration, and the early placement of rituximab therapy may have facilitated the good response.

## 1 Introduction

IgG4-related disease (IgG4-RD) is an emerging immune-mediated disease. From 2003 to nowadays, a common pathogenetic basis has been recognized for dozens of conditions previously considered as unique entities, changing their management.^[1]^

IgG4-related disease can involve almost every tissue, leading to enlargements, fibrosis, and organ dysfunction.^[2]^ Recently, a syndrome known as adult-onset asthma and periocular xanthogranulomas (AAPOX), originally described as case series of 6 patients^[3]^ with asthma and xanthogranulomas, characterized by giant cells, lymphocytic, and plasma cells infiltration, has been included under the umbrella of IgG4-RD.^[4–6]^

Despite accumulating evidence of effectiveness of therapeutic target molecules, corticosteroids remain the first-line treatment of IgG4-RD and AAPOX syndrome.^[7,8]^ Current approach is based on relevant doses of steroids (0.6 mg/kg/d) for 2 to 4 weeks, followed by a slow tapering.^[9]^ This protocol exposes patients to significant risk of relapses and side effects. Steroid-sparing agents, as synthetic immunosuppressive agents (e.g., mycophenolate mofetil, methotrexate, or azathioprine) or rituximab proved to be of benefit in not randomized studies.^[10–12]^

However, monotherapy with rituximab as first-line treatment, in order to totally avoid steroid exposure and reduce relapses, has been attempted in very few cases with specific contraindication (i.e., tuberculosis).

We report the first case of a patient with IgG4-related AAPOX syndrome treated with rituximab as first-line therapy, without steroid, showing an excellent and sustained clinical and laboratory response.

The article has been written according to the International Guideline for case-reporting CARE (http://www.equator-network.org/reporting-guidelines/care).

## 2 Case report

**Figure 1:**
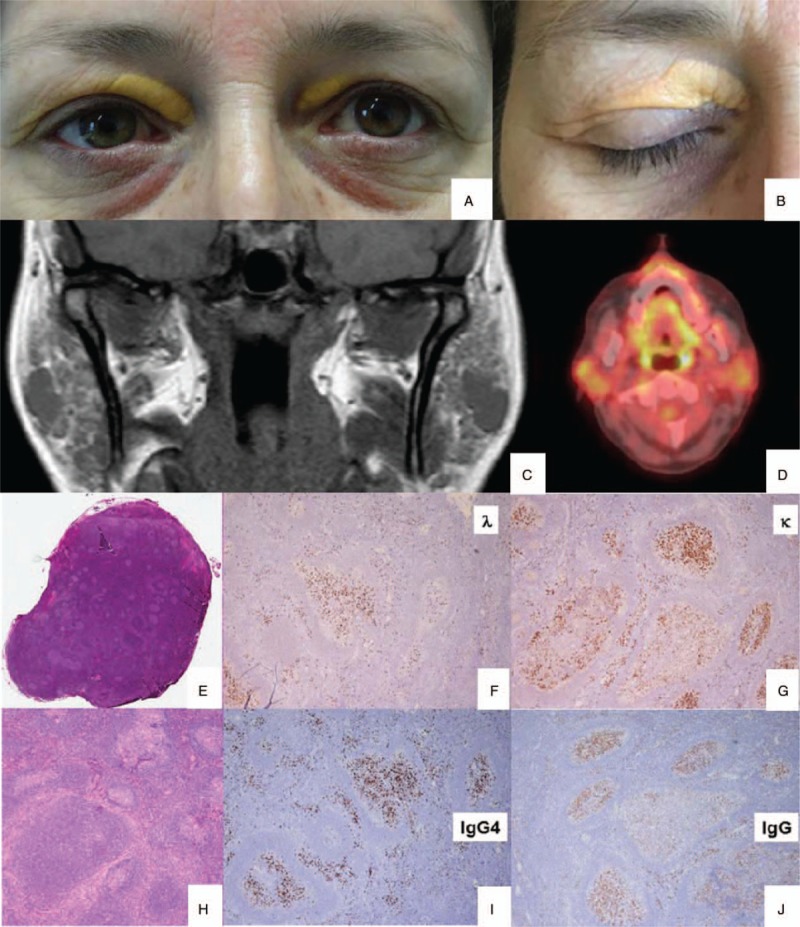
Clinical baseline findings: enlargement of salivary and lacrimal glands and bilateral yellow xanthelasma of eyelids (A, B). Magnetic resonance imaging (MRI) (C) and positron emission tomography (18FDG-PET-CT) (D) baseline images: patchy diffuse salivary and lacrimal glands enlargement with multiple intra-glandular lymph nodes. Histologic appearance of the lymph node (E, H): multiple follicles with variable morphology from normal hyperplastic germinal centers to progressively transformed ones. Immunostainings (F–J): germinal centers and interfollicolar areas rich of plasmacells with polytypic reaction for kappa and lambda light chains (G, F) and high reactivity for IgG (J); the IgG4/IgG ratio is >40%. In immunostainings for CD20 and CD3 lymph node architecture is preserved (not shown).

**Figure 2:**
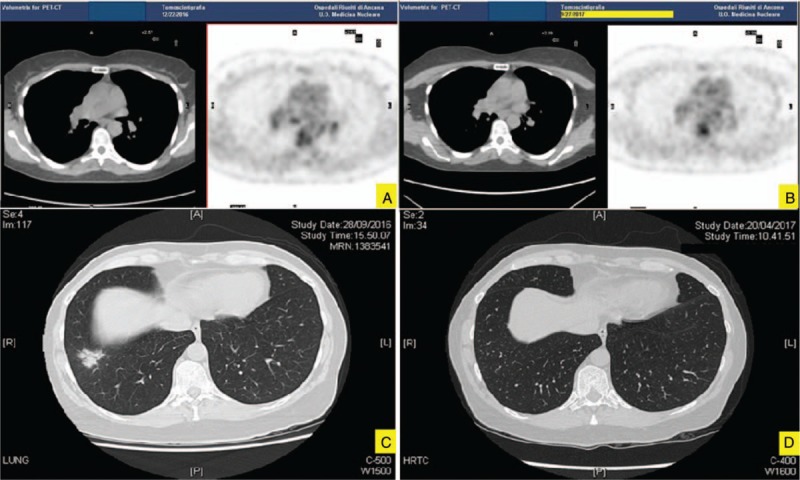
Positron emission tomography (18FDG PET-CT) and HRCT images before (A, C) and after (B, D) a single course of rituximab: attenuation of 18-FDG uptake in lymph nodes and resolution of the nodular lesion at the inferior right pulmonary lobe at 12 months follow-up.

**Figure 3:**
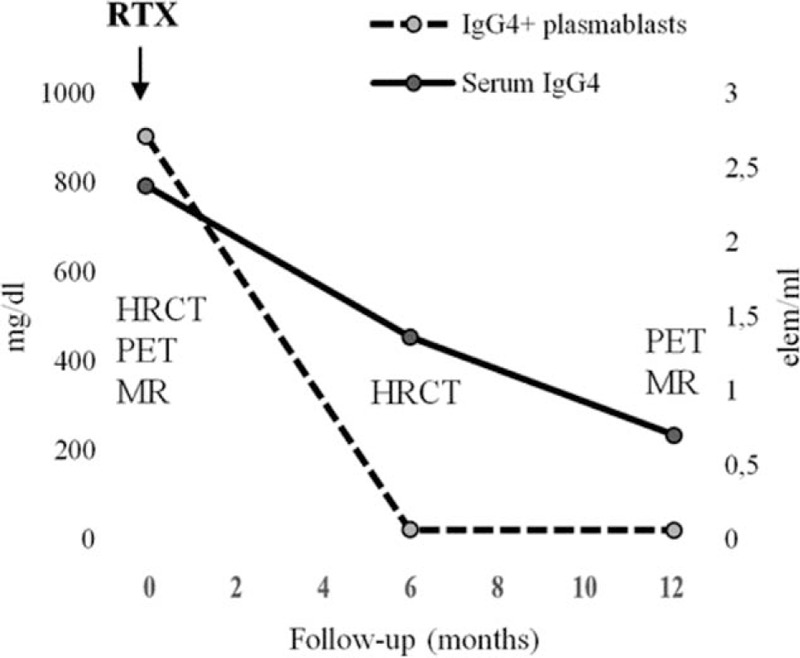
Patient's disease course. HRCT = high resolution computed tomography; MRI = magnetic resonance imaging; PET = positron emission tomography; RTX = rituximab single course (2 g).

A 50-year-old woman was admitted to our hospital on October 2016 complaining of sicca syndrome, slight pain, and bilateral enlargement of parotid glands for 18 months; afterwards, a progressive bilateral periorbital swelling began. Patient did not report fever, cough, or any respiratory symptom. In 2015, Sjogren syndrome was diagnosed, according to clinical and histopathological American College of Rheumatology/European League Against Rheumatism classification criteria.^[13]^ Nevertheless, serum anti-nuclear, anti-SSA/Ro, anti-SSB/La antibodies and rheumatoid factor were persistently negative. Intermittent courses of low-doses steroids (prednisone 5–12.5 mg/d) and hydroxychloroquine 200 mg/d were ineffective. Therapy had been stopped 6 months before our observation due to inefficacy. In her past medical history a moderate asthma for few years, requiring treatment with long acting beta2 agents and inhaled steroids, was noteworthy. Physical examination revealed evident swelling of salivary and lacrimal glands and bilateral yellow eyelids xanthelasmas (Fig. 1A and B). In the suspicion of an underlying indolent lymphoproliferative disease a magnetic resonance imaging (MRI) exam was performed, showing patchy diffuse salivary and lacrimal glands enlargement together with the presence of multiple intra-glandular lymph nodes, without focal lesions (Fig. 1C). Moreover, a high-resolution computed tomography (HRCT) scan of the chest revealed a nodular lesion (diameter 3 cm) at the inferior right pulmonary lobe, with irregular margins and air bronchogram in the context (Fig. 2C). Bronchoalveolar lavage did not show pathogenic microorganisms, nor atypical cells; mixed mononuclear population (macrophages and lymphocytes) emerged at microscopic examination (quantitative analysis not done). An endoscopic biopsy failed to obtain diagnostic material, due to the scarce opacity of the lesion at the fluoroscopic guide. A whole-body positron emission tomography (18FDG-PET-CT) showed a modest increase in fludeoxyglucose up-take of lymph nodes (right axillary [SUVmax = 2.0], right upper paratracheal [SUVmax = 3.1], Barety loggia [SUVmax = 2.8], and subcarinal [SUVmax = 4.5]), parotid glands, and oral-rhino pharyngeal mucosa (Fig. 2A). Laboratory findings confirm the absence of serologic markers of connective tissue diseases, as well as signs of systemic inflammation. Serum IgG4 level was high, almost 4 folds UNL (794 mg/dL). Peripheral blood flow cytometry showed elevated CD19+ CD38^bright^ CD27+ CD20- plasmablasts concentration (2713 cells/mL). Complete laboratory features are summarized in Table 1. Histological examination of an intra-parotid lymph node confirmed clinical hypothesis of IgG4-related disease (Fig. 1E–J), according to the comprehensive diagnostic criteria 2012.^[14]^ After microbiological screening and informed consent, 1 cycle of 2 g rituximab (RTX) (1000 mg each 15 days apart together with methylprednisolone 100 mg single shot premedication) was administered. No chronic therapy was prescribed and no adverse effects were observed. Patient referred a rapid and complete improvement of exocrine glands swelling, and resolution of asthma, allowing bronchodilator therapy discontinuation, whereas xanthelasmas were still visible and unchanged. Remission persisted at the 12-months follow-up visit and was confirmed by MRI. Furthermore, neither the pulmonary lesion was visible at HRCT (Fig. 2D), or significant intra-thoracic 18FDG-uptake was detectable at PET control (Fig. 2B). IgG4-RD responder index^[15]^ (without IgG4 serum level) stepped down from 14 to 6, serum IgG4 levels roughly halved and plasmablasts concentration became undetectable (Table 1, Fig. 3).

**Table 1:**
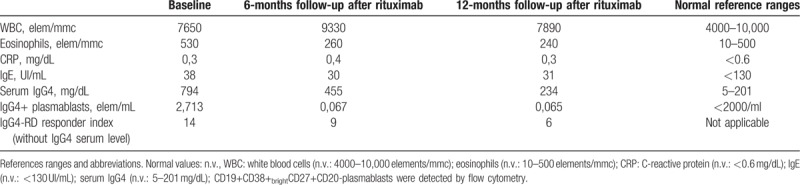
Laboratory data of the patient.

## 3 Discussion

**Figure 4:**
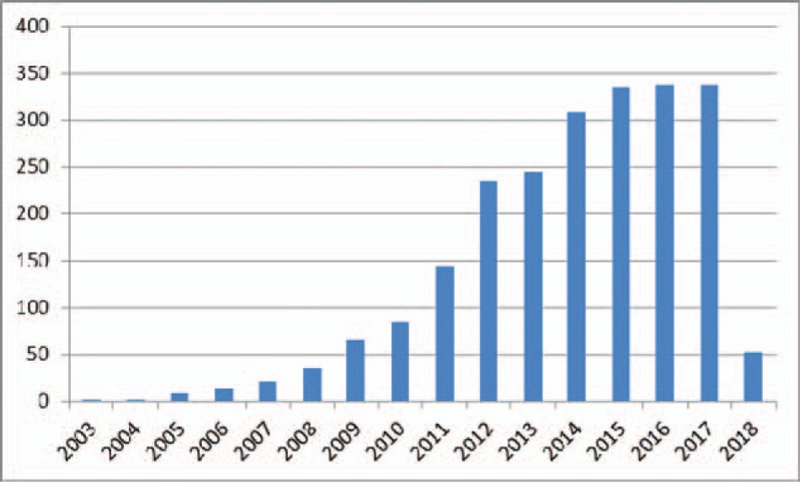
Yearly number of PubMed citations since 2003 containing the search term: IgG4-related (all fields).

From 2003, when IgG4-RD has been proposed as a distinct clinical entity, an increasing number of reports, clinical studies, and reviews on this topic has been published (Fig. 4), improving knowledge about biology, clinical management, and prognosis of this disease.

In first reports, only a variant of sclerosing autoimmune pancreatitis with elevated IgG4 serum levels was included in this definition. Thereafter, it has been expanded to contain conditions characterized by multi-organ involvement and variable clinical behavior, ranging from severe manifestations to oligosymptomatic or even asymptomatic patients. Indeed, IgG4-RD may potentially affect any organ/tissue, such as lacrimal and salivary glands, lymph nodes, orbital or periorbital tissue, breast, skin, pancreas, biliary tract, thyroid, mediastinum, lungs, pleura, pericardium, aorta, arteries, retroperitoneum, kidneys, prostate, pachymeninx, hypophysis.^[16,17]^

Our patient fulfilled all IgG4-related disease comprehensive diagnostic criteria, formulated in 2011 after the first International Symposium of IgG4-RD (Boston 2011), where this pleiotropic disease was recognized as a systemic entity.^[18]^ In addition, the history of adult onset asthma and the presence of prominent xanthelasmas allowed us to diagnose the sixth case of confirmed association between IgG4-RD and AAPOX syndrome.

Pulmonary lesions have not been described in AAPOX population so far. The lack of histological demonstration of tissue infiltration by IgG4 plasmacells makes attribution of observed lung nodule to the IgG4-RD uncertain. However, radiological appearance as solid nodular lesion, that is the most frequent pattern described in IgG4-RD,^[19]^ complete response to the treatment, absence of neutrophils, and microorganisms in the bronchoalveolar lavage are elements of suspicion.

According to the standard therapeutic approach, all previously described AAPOX patients were treated with corticosteroids, mostly at high doses (1 mg/kg/d); in 3/5 cases low-dose methotrexate (2 cases) or rituximab was added due to relapse during steroid tapering.^[5,6]^

In our patient, in order to avoid potentially relevant steroid related side effects in a post-menopausal woman, we administered a RTX monotherapy. Remission of clinical signs and symptoms (lacrimal and salivary glands enlargement and asthma), disappearance of lung nodule, clear attenuation of 18-FDG uptake in lymph nodes followed the 2 g RTX single course. Only xanthelasmas remained unchanged. Remission persisted at the latest follow-up evaluation (>1 year).

RTX for IgG4-RD showed evidence of efficacy in observational cohorts, when used in second-line regimens after steroid failure or to maintain remission. Two interventional clinical trials investigating RTX-steroid association are actually under way (NCT01584388, NCT02458196).

RTX mechanism of action in IG4-RD is still under investigation, despite progresses achieved in understanding disease pathophysiology. Recent data highlight the pivotal role played by follicular helper T-cells (Tfh) infiltrating target tissues and lymph nodes.^[20]^ These cells are responsible for inducing the differentiation of B cells into plasmablasts producing IgG or IgG4 both in the germinative center of lymph nodes and in follicular aggregates in affected tissues. Oligoclonal expansion of CD19+ CD20- CD38^bright^ plasmablasts characterizes peripheral blood of these patients and correlates with serum IgG4 levels. Moreover the proportion of circulating plasmablasts, as well follicular helper T-cells, decreases when clinical remission can be achieved, re-increasing during relapse.^[21,22]^ Therefore, flow cytometry may represent a useful tool for disease activity monitoring. Although CD20 negative, plasmablasts concentration is clearly affected by RTX treatment, maybe through depletion of CD20+ progenitors pool.^[22]^

In our patient circulating number of plasmablasts was higher than usually measured in healthy population.^[20,21]^ However, in comparison to available data from IgG4-RD case series, plasmablasts concentration was located in the lower part of the range, reflecting the limited extension of systemic involvement. This finding may contribute to explain the surprisingly deep and persistent response to treatment, as seen in other immune-mediated diseases, where plasmablasts baseline level predicts response to rituximab.^[23]^

IgG4-RD is a complex disease with high variability in clinical and biological behavior. A better understanding of underlying molecular mechanisms may contribute to improve risk stratification and could lead to a tailored treatment selection and monitoring.

## Acknowledgments

The authors warmy thank Dr Luca Butini for performing flow-cytometric analysis, Dr Giuseppe Garraffa, and Dr Fabio Fringuelli for their support to CT-PET scan interpretation and analysis.

## Author contributions

**Conceptualization:** Giovanni Pomponio, Diletta Olivari, Massimo Mattioli, Alessia Angeletti, Giulia Rossetti, Gaia Goteri, Armando Gabrielli.

**Data curation:** Giovanni Pomponio, Diletta Olivari, Massimo Mattioli, Alessia Angeletti, Gaia Goteri, Armando Gabrielli.

**Formal analysis:** Giovanni Pomponio, Diletta Olivari, Massimo Mattioli, Gaia Goteri, Armando Gabrielli.

**Funding acquisition:** Armando Gabrielli.

**Investigation:** Giovanni Pomponio, Massimo Mattioli, Gaia Goteri, Armando Gabrielli.

**Methodology:** Giovanni Pomponio.

**Writing – original draft:** Giovanni Pomponio, Diletta Olivari, Massimo Mattioli, Gaia Goteri, Armando Gabrielli.
